# Physicians’ Perceptions of the Quality of Palliative Care and of the Importance of Collaboration in Interdisciplinary Teams in Norwegian Nursing Homes

**DOI:** 10.2147/JMDH.S487153

**Published:** 2025-01-25

**Authors:** Liv Skomakerstuen Ødbehr, Reidun Hov, Harald Sanaker, Tuva Sandsdalen

**Affiliations:** 1 Department of Health and Nursing Sciences, Faculty of Social and Health Sciences, University of Inland Norway, Elverum, Norway; 2 Centre for Development of Institutional and Home Care Services Inland (Hedmark) Norway, Hamar Municipality, Norway; 3 Municipal Medical Center, Ringsaker Municipality, Brumunddal, Norway

**Keywords:** palliative care, nursing homes, physicians, end-of-life, health personnel

## Abstract

**Purpose:**

The palliative phase of a patient’s life is often characterized by disease complexity, increasing the need for holistic care, support for the patient’s relatives, and the up-to-date knowledge of a multidisciplinary healthcare team. Physicians in nursing homes have the main responsibility for providing palliative care to vulnerable and fragile patients. There is limited research uncovering physicians’ experience and perceptions of what is important in this phase of patients’ lives. The aim of the study was thus to investigate physicians’ perceptions of factors that influence the quality of palliative care in Norwegian nursing homes and their descriptions of the importance of the team’s collaboration.

**Materials and Methods:**

The study has a qualitative research design based on interviews with twelve nursing home physicians in Eastern Norway. Interviews were conducted between February 2023 to May 2023, analyzed using qualitative content analysis.

**Results:**

Three themes describe the content of the findings: i) Comprehensive care plans enhance the quality of palliative care, ii) A collaborative team provides higher-quality care than the sum of its parts, iii) Systemic and environmental factors affect the ability to ensure continuity of care.

**Conclusion:**

The physicians in this study expressed that the quality of palliative care in nursing homes depended on comprehensive care plans, including up-to-date knowledge of medical treatment options, partnership with the patient and relatives, and a consistent holistic approach to the patient. The quality also depended on the interdisciplinary team’s collaboration in assessing the patient, observing symptoms, and planning further care and treatment in accordance with patients’ and their relatives’ preferences and wishes. Finally, systemic and environmental factors affected the ability to ensure continuity of care. Further work is needed to ensure that systemic factors enable physicians to deliver high-quality palliative care and that a comfortable physical environment is created in nursing homes.

## Plain language summary

In this study, we interviewed twelve nursing home physicians and asked them about their views on the quality of palliative care in nursing homes and what they thought about the importance of working in interdisciplinary teams to provide the best possible care for patients. The interviews were conducted face-to-face at the physicians’ workplace. All interviews were recorded on audio files, transcribed and analyzed using content analysis. The physicians said that they valued cooperating and exchanging expertise in an interdisciplinary team. As they had little time available for each patient, the team’s efforts were invaluable. The quality of care depended on good observations by healthcare professionals, as well as follow-up actions. The quality was good when the collaboration worked. Also, important for maintaining good quality were different aspects of the healthcare system, and a suitable physical environment that made patients and relatives feel cared for. The findings of this study show that there is a need for interprofessional expertise and that each member of the team is important for the best possible outcome. Good cooperation depends on openness and respect, especially in difficult issues where there are disagreements or different views on how to solve a particular case. This study shows that good and sufficient resources improve care, especially when the interdisciplinary team works well together.

## Introduction

Palliative care (PC) is an approach that aims to provide optimal symptom relief and quality of life for patients with a serious or life-threatening illness.^1–3^ Palliative care should be individually adapted, person-centred, involving an ethical responsibility for the healthcare providers, regardless of the patient’s age, diagnosis and functional level.^4^ It requires a holistic understanding of how to relieve pain and suffering through early identification.^1^,^3–6^ Knowledge of the patient’s underlying disease and previous treatments, systematic symptom assessment, and collaboration across levels of care are key factors in palliative care.

Quality in palliative care therefore implies comprehensive care adapted to the individual patient’s needs, whether these are physical, psychological, social, or existential.^4^,^7–11^ End-of-life often involves a complex condition, which increases the need for holistic care, support for relatives, and up-to-date knowledge among healthcare professionals.^1^,^12^ Conversations with patients about treatment to improve their prognostic awareness and provide them with coping strategies will take time, but they are particularly important for patients who are afraid of what lies ahead.^5^ This implies helping patients to manage their emotional reactions in the end-of-life trajectory.^5^,^13^ Existential maturation processes show individual variation. Many patients will find it difficult to understand and absorb the prognosis of the disease and to express their preferences for the palliative care. Healthcare professionals must take patients’ worries into consideration, and good communication skills are required to manage patients’ preferences and wishes.^12–15^ That includes engaging patients and their relatives in planning and decisions on care plans.

Norwegian nursing homes (NHs) are municipal residential care facilities for people with extensive and often long-term healthcare needs. Most patients in Norwegian NHs receive comprehensive inpatient care around the clock. Statistics show that more than half of all deaths in Norway every year occur in NHs, which in 2019 constituted around 16,300 people.^16^ Thus, palliative care predominates in NHs.^16^ The majority of these patients have multiple comorbidities. In Norway, they are mostly above 70 years of age, and their average lifespan in an NH is about two years.^17^ Many patients in NH suffer from cognitive decline, such as dementia, which affects their capacity to make decisions at the end-of-life, and they are thus dependent on relatives or the NH staff to promote their needs and wishes.^3^,^18–20^ General expertise, knowledge and skills in palliative care as well as good communication and shared understanding of the patient’s situation are necessary to achieve satisfactory results.^12^

Physicians working in Norwegian NHs have expertise in various specialties in their field and have the primary responsibility for the medical treatment of patients.^1^,^12^ Most physicians have part-time positions in the NH and are thus dependent on the interdisciplinary care team’s observations to obtain rich and nuanced information about patients’ condition, due to the complex symptom burden of most patients.^21^ Interdisciplinary teams may include eg, physicians, nurses, a physiotherapist, an occupational therapist, social workers, and a priest.^22^ The physicians work closely with the other professionals, maintain a dialogue with the patients’ relatives, and cooperate on various levels in the healthcare system.^23^ In many parts of Norway, few clinicians are available, and resources are scarce, while the available staff feel unsure about how best to provide palliative care.^6^,^7^,^9^,^22^,^24^ Research reveals some barriers to maintaining high-quality palliative care, such as the difficulty for many physicians and nurses to find the right moment to start end-of-life care.^2^ Further, physicians and patients are apparently not always concerned about the same issues in terms of what constitutes quality in palliative care.^2^,^25^,^26^ Other problems are linked to healthcare professionals’ limited training in palliative care.^23^ This can place a greater responsibility for comprehensive care on physicians, who have limited time to fulfill their duties in nursing homes. Furthermore, although physicians collaborate with other professional groups, they still have the formal responsibility for ensuring that the overall symptom relief is good. There is a need for more research to explore physicians’ perceptions of palliative care in terms of strengths and weaknesses of patient care in NHs and the importance of the professional care team and its responsibilities to maintain good quality. The aim of the present study is thus to investigate physicians’ perceptions of factors that influence the quality of palliative care in Norwegian NHs and their descriptions of the importance of collaboration in the interdisciplinary team.

## Materials and Methods

This study has a qualitative research design based of individual interviews with twelve NH physicians. The qualitative method was adopted because it was the most suitable method for obtaining the physicians’ perceptions of palliative care.^15^,^27^ Two articles are developed based on the same data material. The first article was published in June 2024 where the aim was to explore how physicians understand and experience advance care planning and follow-up of care plans in Norwegian nursing homes. Data in that study was generated from the second part of the interview guide.^28^ The current article is the second one, however, and aim to investigate physicians’ perceptions of factors that influence the quality of palliative care in Norwegian nursing homes, and their descriptions of the importance of the interdisciplinary team’s collaboration. Data in the present study is essentially generated from the first part of the interview guide and refers to several factors that affect the quality of palliative care beyond advance care planning. The study adhers to consolidated criteria for reporting qualitative research checklist (COREQ) (Supplementary material 1).

### Setting and Recruitment of Participants

Informants were recruited from the network of NH physicians in Eastern Norway, the Centre for the Development of Institutional and Home Care Services in Inland County, using purposive sampling.^28^,^29^ The physicians represented different NHs from several municipalities in Inland County. It was preferable to include NH physicians rather than GPs as NH physicians follow the patients more closely. The medicine program includes education in palliative care on a general basis which means that all informants had a basic knowledge of PC.

### Inclusion Criteria

Information about the study was given verbally at the network meeting with a request for participation into the study. Twelve physicians agreed to participate and were included in the study. The main inclusion criterion was that the physicians had experience from palliative care in the NH where they worked. Physicians with little or no experience with palliative care in NH were excluded from the study. The participants consisted of both male and female physicians of varying ages. The variation was evident in the physicians’ different ethnicities, how long they had worked after their education and how long they had worked as NH-physicians. Half of them had studied medicine in other European countries.^28^ Some worked part-time in the NH, while others worked full time. Several of the physicians also worked as GP. Most had extensive experience from working in hospitals, and some were specialists, in, eg, geriatrics or general medicine. The size of the nursing homes where the physicians worked varied from six patients to eighty. There were palliative patients in all the nursing homes, and some also had palliative units.^28^ The background and characteristics of the participants are shown in Table 1.

**Table 1:** Background and Characteristics of Participants

		n	Range
Gender	Male	8	
	Female	4	
Age (yrs)	23–35	3	23–64
	36–50	5	
	51–65	4	
Country of education	Norway	6	
	Other European countries	6	
Highest qualification	Medical doctor	9	
	Specialist training and/or PhD	3	
Work experience (yrs)	2–10	4	2–36
	11–20	2	
	21–30	1	
	31–36	3	
	Missing	2	
Work experience in nursing homes (yrs)	1–10	8	1.0–30
	11–20	3	
	21–30	1	
Percentage of full-time job in nursing home	20	2	20–100
	25	1	
	40	2	
	100	7	

### Data Collection

Data were collected between February 2023 to May 2023. Information about the study was communicated orally to the physicians at a network meeting and by Email with supplementary information. The interviews were conducted by an experienced nurse (female, PhD) and a physician (male MD) from the research group at the NH where the physicians worked. One of them led the interview, while the other took notes along the way. The notes were an aid to ask follow-up questions. The experience from the interview situation was that the topic was engaging and that the interviews proceeded as a mutual dialogue. Each physician was interviewed once. No one dropped out. The interviews lasted between thirty and sixty minutes and were recorded on audio files, transcribed verbatim to written text and then analyzed. No qualitative data analysis software was used (Supplementary material 2).^29^ Examples of the interview questions are shown in Table 2.

**Table 2:** Examples of Questions From the Interview Guide

**Q1:**	What does good palliative care mean to you?
**Q2:**	Which factors promote and inhibit the possibility to provide palliative care in nursing homes?
**Q3:**	What do you think characterizes good interdisciplinary collaboration?

### Analysis

Analysis of the transcribed text was based on Graneheim and Lundman’s^27^ qualitative content analysis. They describe the analysis as a process that involves a back-and-forth movement between the whole and parts of the text. Key concepts in the analysis are manifest and latent content. The transcribed interviews were read through several times by all members of the research team to gain a general idea of the content in the entire data set. The manifest content is what the text says, and the text was i) divided into small meaning units such as words or statements that related to the same meaning and then ii) condensed by shortening the meaning units while still preserving the same content. That meant giving a summary description and interpretations on a higher logical level, and the meaning units were thereafter iii) sorted into codes, which enabled the text to be thought about in a different way. The various codes were then assessed based on similarities and differences and iv) sorted into sub-categories, which were reflected on and formulated as precisely as possible, before being sorted into main categories. The categories were v) described as exhaustive and mutually exclusive. Tentative categories were discussed by the research team, and decisions were made on whether or not to include them. The research team then discussed all the codes and categories until a common understanding on their structuring was reached. This constituted the manifest content of the text.

The visible and obvious content in the text was thus revealed in the categories, while the latent meaning in the text was formulated in terms of themes. To formulate sub-themes and themes involves an interpretation of the underlying meaning of the text, depending on the reviewer’s subjective interpretation, which we also took into consideration. Examples of steps in the content analysis are shown in Table 3.

**Table 3:** Overview of themes and sub-themes

Sub-themes	Themes
Pain relief Clarify the patients’ needs Involve the patient’s relatives	Comprehensive care plans enhance the quality of palliative care
Each team member counts Complementary information of high value	A collaborative team provides higher quality than the sum of its parts
Documentation systems Physical surroundings	Systemic and environmental factors affect the ability to ensure continuity of care

## Results

The terms informant and physician are used interchangeably in the presentation of the findings. The manifest content of the analysis in the form of subcategories and categories is presented under each sub-theme and main theme. The latent content revealed that physicians’ perception of the quality of palliative care in NHs was linked to the follow-up of the patients’ symptom relief among others. They were thus dependent on the fact that the healthcare professionals' work was based on a holistic care approach. Three themes were developed to represent the physicians’ perceptions of factors that influence the quality of palliative care in Norwegian NHs and their descriptions of the importance of collaboration in the interdisciplinary team: i) Comprehensive care plans enhance the quality of palliative care, ii) A collaborative team provides higher-quality care than the sum of its parts, iii) Systemic and environmental factors affect the ability to ensure continuity of care.

### Comprehensive Care Plans Enhance the Quality of Palliative Care

This theme is described from three perspectives that emerged clearly in the results, providing symptom relief, holistic care plans and involving patients and their relatives.

#### Provide symptom relief

Comprehensive care that included medical treatment plans which could also bring pain relief to patients was considered by the physicians to improve the quality of palliative care in the NHs. The physicians emphasized pain and symptom relief as a core issue in palliative care. They highlighted that palliative care differs from other medical treatment by being non-curative, but aimed at relieving unpleasant symptoms that dominate the patient’s daily life. They stated that this focus came from experience. One of the informants said:

Palliative care is that everyone should feel relief and feel as well as possible, and that we should help them with their pain and unpleasant symptoms in the best possible way. (Informant 11)

Several of the physicians mentioned that they followed both national and international guidelines in planning and implementing palliative care for patients. They emphasized being at the forefront to ensure high quality in patient care. When they talked to some of the patients, it was also usual to explain current research and up-to-date knowledge in the field. One of the informants pointed out that he emphasized an evidence-based focus on the treatment offered:

I try to get information from patients about what they really want, what their expectations are for their stay and further plans. I also try to provide good information about prognoses, statistically speaking, and what to expect and to what extent. So I always ask if they are curious about anything. I also try to explain how the system works, how we work on the ward, and on which day the physicians do their rounds in the nursing home. (Informant 8)

The time before the palliative care commenced was important in the patient’s life, as well as the transition from the phase of curative treatment to the palliative phase. The physicians felt that it was important to know the patients’ background and history to provide optimal care during this period of their life. They emphasized that it was vital to observe the patients for a while before they could give them individualized care.

…you talk more about interpersonal issues because you have had time to get to know the person in question. Then you can ask the right questions, you see, because you know a bit about who that person is. Then it is easier to talk about medical history and what is important to that person when the end of life is approaching. (Informant 3)

The physicians felt that they had to find a balance in their ambitions to provide care. The right level of medical treatment helped patients to participate in daily life as much as possible. Regular follow-up was key to maintaining continuity of care.

#### Holistic care plans

The physicians described palliative care as creating a dialogue with the patient, listening to the patient and relatives and clarifying the patient’s needs and wishes. Dialogue enabled patients to express what was important to them and empower them to make informed choices. An important way of ensuring this was by physicians showing respect and establishing a trusting relationship with patients through honest talk. They emphasized being able to understand the psychological mechanisms of patients to convey a feeling of security, especially when talking about the disease and end-of-life treatment. An important goal was to reduce anxiety in patients’ lives, be sensitive and display trust and understanding of the patient’s grieving process. Planned meetings and conversations about end-of-life and dying were considered a suitable starting point for a dialogue and reflection about different alternatives to treatment and end-of-life care. One informant said:

It might be wise to be a little more diplomatic, and not be too harsh by saying that you’re going to die but explain that it’s natural for all people to end one day. We just don’t know exactly when it will happen. (Informant 4)

Some tension between patient and physicians could sometimes arise in the dilemma of being realistic and maintaining hope in questions of end-of-life care. The physicians tried to explain clearly the reasons for their suggestions and the different priorities regarding treatment and care in such cases. It was important not to push or force patients in any way. The physicians stated that experiential knowledge in palliative care was important and that it was acquired over time. They felt that a lack of humility in relation to patients and relatives was always a mistake and emphasized maintaining a positive attitude even when they were challenged by dissatisfied patients.

#### Involving patients and their relatives

The patients’ relatives were seen as an important resource for most patients as they speak on their behalf. The physicians emphasized that it was important to involve them in care plans. The sense of a lack of autonomy was difficult for both patient and relatives. The physicians were aware of this, and they always tried to include those who were close to the patient in decisions. One of the informants explained:

Many of them need to have relatives with them a lot, and it is essential to ensuring that the patient’s needs are met. Some patients don’t think about it or don’t feel that they can express their own wishes and needs. That is why it’s very useful for the patient to bring the relatives. (Informant 1)

In their work, the physicians had to make tough decisions. On occasions, they could find this difficult, especially if the patients’ relatives had different views on the care and treatment. Poor communication sometimes affected collaboration between relatives and clinicians. When communication was restored, they usually discovered the misunderstandings or other reasons why communication had broken down.

### A Collaborative Team Provides Higher Quality Care Than the Sum of Its Parts

The physicians were concerned about the importance of collaboration with other professional groups. The findings are described from two perspectives: Each team member counts and to be included in the theme.

Each team member counts

The physicians appreciated the fact that a team provided better care than the sum of its parts. This strength was dependent on each team member showing autonomy in their area of competence and responsibility. Well-coordinated teams meant high-quality palliative care. The physicians felt that they were dependent on the observational skills and information of the other professionals in the healthcare teams. One of them described it as follows:

I like the interdisciplinary meetings where the physiotherapist, specialist nurse and the patient’s regular nurse sit together and plan. I like to prioritize those meetings, because I get a lot of information that I might not necessarily get otherwise. Then everyone sits together and can discuss thoughts about current and future treatment. (Informant 9)

Nurses who had considerable experience in palliative care provided support to the physicians, who often found that nurses understood the situation in the same way as themselves. This was perceived as enhancing the quality of palliative care, and the physicians noticed that continuity of care and treatment made patients feel secure.

To be included in the team

The physicians also obtained constructive and complementary information of high value from other professional groups, as one of the informants highlighted.

A good interdisciplinary team is essentially a team that can take care of all the patients’ needs. By that I don’t mean only the medical side, but also care, and particularly mobilization and rehabilitation. That’s why it’s a good idea to have a physiotherapist, a nurse, healthcare workers and physicians in the nursing home. (Informant 10)

Quality could be compromised in situations where the other professionals wanted to lighten the physicians’ workload. Some healthcare workers could exclude the physicians from meetings altogether, and the physicians then felt like visitors to the ward rather than part of the care team. Other negative factors for quality care were healthcare workers who lacked enthusiasm and interest in their work or who were new and very insecure. Some healthcare workers did not want the physician to pass on their expertise, which could be perceived as exclusionary and dismissive. The physicians pointed out that if the team feeling was lost, it might often be due to a lack of goodwill or motivation. They had found that this was rare, but they had occasionally noticed it through their many years of experience.

### Systemic and Environmental Factors Affect the Ability to Ensure Continuity of Care

Structural factors in departments, healthcare systems, and service organization played a role in patient care. The physicians expressed that good work routines were helpful in maintaining quality in palliative care. Sufficient time and available resources were mentioned as important by several of them. They had many tasks to perform in palliative care despite already having a very high workload. The physicians said that the documentation system as well as physical surroundings affected the continuity of palliative care.

#### Documentation systems

The documentation system was a topic that several of the physicians mentioned. It was a familiar challenge to access information and find relevant information. The documentation systems were not always compatible, and they spent a great deal of time searching for up-to-date information about patients. They meant that this was time-consuming and unnecessary, and they hoped for changes in the future. One informant explained:

We have talked a little about how to ensure that information is clearly communicated to everyone. One aspect is the communication between day and night shifts. Another aspect is when patients are transferred to home. (Informant 2)

#### Physical surroundings

Physical surroundings were mentioned by the physicians as important for good patient care. They emphasized that there had to be suitable private rooms available to prevent important dialogues with patients from taking place in the hallway or in open rooms in the NH. They also experienced that it was not necessarily appropriate to talk about certain topics in the patient’s room. Pleasant surroundings and attractive colors on the walls in the patient rooms had been mentioned by some patients, and the physicians therefore meant that colors and aesthetics were important aspects of treatment and care for seriously ill people. They saw this as part of making patients feel comfortable and easing their burdens.

## Discussion

The aim of this study was to investigate physicians’ perceptions of factors that influence the quality of palliative care in Norwegian nursing homes and their descriptions of the importance of collaboration in the interdisciplinary team. The main findings reveal that the quality of palliative treatment and care is enhanced when healthcare professionals have up-to-date knowledge and broad expertise in comprehensive palliative care and suitable care plans. Furthermore, the quality of palliative care depends on the ability to utilize the combined expertise and arrive at a common understanding of the content of patient care and treatment. The care provision system and environment must also be better customized to care for patients and their relatives in the end-of-life situation. This study shows that the quality of care varies between the different NHs and that there is a need for more knowledge and competence in PC among healthcare professionals. This study also reveals that even though the physicians followed the guidelines, the quality of care was dependent on the team’s overall competence.

Quality in palliative care is first a question of good care strategies in terms of ensuring patients’ sense of trust and meeting their needs for social relationships and self-esteem throughout their illness.^11^,^30^,^31^ Second, it means ensuring up-to-date knowledge on medical treatments and symptom relief, in line with recognized guidelines for palliative care.^4^,^12^,^32^ The physicians in this study were concerned about this and wanted to follow international standards for palliative care.^4^ One area that they emphasized was the transition from curative treatment to palliative care, which is a difficult phase for many patients.^33–35^ They highlighted this because patients in NHs are vulnerable and suffer from comorbidities and cognitive impairment, which prevents them from communicating freely about their needs and wishes.^15^,^33^,^36^,^37^

The physicians also emphasized meeting patients’ needs for consistency and self-determination, respecting patients’ choices, and showing openness and humility regarding patients’ wishes. Sometimes this was difficult due to communication breakdowns or lack of time, as other studies show.^38^ On the other hand, quality is achieved when healthcare professionals are available for patients and can enable them to participate and influence care.^34^ The findings are well aligned with international standards for good palliative care.^20^

Quality in palliative care also includes supporting patients and their relatives who experience loss and grief. The physicians in this study found it important to maintain a respectful dialogue with the patient and relatives, which several other studies also emphasize as vital in end-of-life care.^32^,^36–38^ Another aspect that the physicians highlighted was that they and the patients may differ in their ideas of quality and their situational awareness, as also seen in other studies.^25^,^32^,^37^ A study from 2019 found that the need to talk about death varied between patients and relatives.^39^ Some avoided the topic, and even the healthcare professionals could be reluctant to provide information on the end-of-life scenario as death may be considered taboo. Patients must be asked the question “What is important to you?” Eg, in advance care planning meetings.

The physicians in the current study emphasized the importance of thinking holistically about all aspects of patients’ lives, including their social and psychological needs. Values such as showing respect were emphasized to fulfill patients’ need for self-esteem and self-worth during the end-of-life process. Research shows that patients often trust physicians’ assessments and resign themselves to decisions made by them because they feel confident.^39^ In summary, the study informants perceived the quality of palliative care in the NH as meeting national and international standards.^20^ They also had a clear perception of strengths and weaknesses in the planning and implementation of palliative care in the NH.

Another area highlighted by the physicians was the need for information from the interdisciplinary team in terms of their observations, advice, and insights from their interactions with patients.^32^,^39^ The quality of palliative care is enhanced when the interdisciplinary team with its various competencies collaborates on plans and strategies for care and treatment in the end-of-life situation.^10^ This study shows that interdisciplinary collaboration is valuable precisely because it provides complementary and broadened expertise that benefits patients and relatives.

Those who have closest everyday contact with patients can most easily facilitate a good dialogue between patient and relatives, and sometimes they helped to restore a weakened dialogue. An important task for healthcare professionals is to inform the people patients want to be present when they die. Another important task is to care for patients and relatives in the grieving phase. These findings concur with those in a study by Barnett et al,^13^ where the authors saw that positive attitudes towards care for the dying helped to reduce patients’ fear of death.

Communication and well-functioning systems that support the work of the staff play a role. This is perhaps the greatest challenge in maintaining continuity and quality in palliative care. Communication and interaction are also mentioned as key issues in other studies.^5^,^32^,^34^,^39^ Sometimes the patient’s relatives are included as part of the interdisciplinary team around the patient. The fact that they know the patient best is therefore a form of quality assurance. Patients and relatives can sometimes have different views on certain issues in the palliative phase. This can often be due to a decline in the patient’s cognitive skills.^18^,^22^ Relatives can be of great assistance in decisions about continuation or discontinuation of treatment, in defining goals and preferences, and in planning care and treatment on behalf of the patient. The WHO points this out in its description of good palliative care with an emphasis on person-centred care.^10^

The present study also shows that physical surroundings matter. The environment can help to ensure that the last days of life are felt to be dignified and respectful by patients and their relatives. The physicians highlighted that systemic and environmental factors affected the team’s ability to ensure continuity of care. Here, the main points were having enough time and available resources to provide high-quality palliative care and documentation systems that ensured access to and communication of essential information. However, these factors were perceived to be insufficient in this study and therefore an obstacle to providing good palliative care for the patients in the NH. The lack of resources, eg, few healthcare professionals available, and how this may challenge the provision of high-quality palliative care has been confirmed in previous studies, which highlight the importance of targeting the above factors through quality improvement initiatives.^6^,^7^,^9^,^24^,^26^

The physicians also emphasized the physical surroundings, such as appropriate rooms for conversations and a safe and comfortable environment, as important for good patient care. The importance of the environment has previously been described as an key factor for patients in palliative care in various care contexts.^40–42^ However, this study contributes by providing physicians’ perspectives on this issue.

### Methodological Considerations

Physicians in NHs may be difficult to recruit due to a very heavy workload. It is therefore considered a strength that 12 physicians participated in this study. All had experience in palliative treatment and care from specialist and municipal healthcare services. Their views were therefore based on a broad background of experience. The semi-structured interview guide was developed by the research team and was based upon knowledge of the palliative care field. The interview guide was then piloted, which led to minor adjustments such as modifications of words. The richness of the interviews was also a strength of the study.

A potential weakness and strength in this study could be that all members of the research team had a healthcare background and clinical experience of palliative care from various care contexts. The research team was aware that their experience might influence the analysis and interpretation of data. To minimize this risk, the analysis was performed by moving back and forth between the transcribed text and the four steps in the analysis to ensure that the findings emerged from the informants’ voices. Furthermore, the need for an awareness of each one’s pre-understanding during the analysis work was discussed in order to be open to the informants’ perspective. Each person’s experience from clinical practice made it possible to understand the context in NH. At the same time, the preconceptions could be a hindrance seeing new aspects of PC. These considerations were discussed in the research group. The steps in the analysis were performed by the research team through joint reflection that led to the generation of the themes. The results were substantiated with quotes.

## Conclusions

The physicians in this study perceived that the quality of palliative care in nursing homes depended on comprehensive care and treatment plans, including up-to-date knowledge of medical treatment options, partnership with the patient and relatives, and a consistent holistic approach to the patient. The quality also depended on the interdisciplinary team’s collaboration in assessing the patient, observing symptoms, and planning further care and treatment in accordance with patients’ and their relatives’ preferences and wishes. Finally, systemic and environmental factors affected the team’s ability to ensure continuity of care. Further work is needed to ensure that systemic factors enable physicians to deliver high-quality palliative care and that a pleasant and comfortable physical environment is created in nursing homes.

## Data Availability

The datasets used and/or analyzed during the current study are available from the corresponding author upon reasonable request.

## Abbreviation

NH, Nursing Home.
